# The Prevalence of Compassion Fatigue and Burnout among Healthcare Professionals in Intensive Care Units: A Systematic Review

**DOI:** 10.1371/journal.pone.0136955

**Published:** 2015-08-31

**Authors:** Margo M. C. van Mol, Erwin J. O. Kompanje, Dominique D. Benoit, Jan Bakker, Marjan D. Nijkamp

**Affiliations:** 1 Department of Intensive Care Adults, Erasmus MC University Medical Center, Rotterdam, The Netherlands; 2 Department of Intensive Care, Medical Unit Ghent University Hospital, Ghent, Belgium; 3 Faculty of Psychology and Educational Sciences, Open University of the Netherlands, Heerlen, The Netherlands; University of Stellenbosch, SOUTH AFRICA

## Abstract

**Background:**

Working in the stressful environment of the Intensive Care Unit (ICU) is an emotionally charged challenge that might affect the emotional stability of medical staff. The quality of care for ICU patients and their relatives might be threatened through long-term absenteeism or a brain and skill drain if the healthcare professionals leave their jobs prematurely in order to preserve their own health.

**Purpose:**

The purpose of this review is to evaluate the literature related to emotional distress among healthcare professionals in the ICU, with an emphasis on the prevalence of burnout and compassion fatigue and the available preventive strategies.

**Methods:**

A systematic literature review was conducted, using Embase, Medline OvidSP, Cinahl, Web-of-science, PsychINFO, PubMed publisher, Cochrane and Google Scholar for articles published between 1992 and June, 2014. Studies reporting the prevalence of burnout, compassion fatigue, secondary traumatic stress and vicarious trauma in ICU healthcare professionals were included, as well as related intervention studies.

**Results:**

Forty of the 1623 identified publications, which included 14,770 respondents, met the selection criteria. Two studies reported the prevalence of compassion fatigue as 7.3% and 40%; five studies described the prevalence of secondary traumatic stress ranging from 0% to 38.5%. The reported prevalence of burnout in the ICU varied from 0% to 70.1%. A wide range of intervention strategies emerged from the recent literature search, such as different intensivist work schedules, educational programs on coping with emotional distress, improving communication skills, and relaxation methods.

**Conclusions:**

The true prevalence of burnout, compassion fatigue, secondary traumatic stress and vicarious trauma in ICU healthcare professionals remains open for discussion. A thorough exploration of emotional distress in relation to communication skills, ethical rounds, and mindfulness might provide an appropriate starting point for the development of further preventive strategies.

## Introduction

An Intensive Care Unit (ICU) can be full of stressful situations for patients, relatives and healthcare professionals. A growing body of evidence suggests that burnout among ICU nurses [1] and ICU physicians [2] is a remarkable result of the demanding and continuously high-stress work environment. It has been suggested that ICU professionals could be emotionally affected by end-of-life issues [3], ethical decision making [4], observing the continuous suffering of patients [5], disproportionate care or medical futility [6], miscommunication [7], and demanding relatives of the patients [8]. Moreover, many patients in the ICU lack decision-making capacity; therefore, the healthcare professionals depend on communication with relatives for decision making, which can complicate the communication process [9]. In addition, the ICU work environment has become increasingly technical, which requires extended skills in advanced life sustaining medical therapies.

These aspects may lead to moral distress or avoidance behavior [10], and consequently increase emotional distress. This job stress can have a negative effect on an individual´s enjoyment of work. It might even result in long-term absenteeism or a threatening brain and skill drain if the professionals leave their jobs prematurely to preserve their own health, ultimately leading to economic burdens [11]. In addition, these processes may even reduce the quality of care for patients and relatives [12].

Work-related stress with the accompanying emotions provoked specifically in ICU is well documented over the previous years [12–15]. The high-stakes, high stress environment that ICU professionals practice in, are incredibly demanding intellectually, physically, and emotionally. Both physical warning signs (such as headaches, sleeping disturbances, low back pain and stomach problems) and mental responses (such as irritability or hostility, loss of concentration, low self-confidence and emotional instability) could indicate individual stress reactions [16–18]. However, these are non-specific symptoms which cannot depict the origin of stress and subsequently constrain effective coping mechanisms or the developing of preventive strategies for this ongoing process.

Stress reactions are the first indication of the presence of an emotional trauma. These reactions are defined as a set of conscious and unconscious behaviors, cognitions and emotions, to deal with the stressor [19]. In the research field of traumatization, which focuses on the process and origin of developing stress symptoms, there is a distinct difference in primary and secondary traumatization [20]. Primary traumatization is the process that can occur from having persistent, intense and direct contact with a traumatic event, such as a situation of war, violence or sexual abuse. This process can lead to posttraumatic stress disorder [21]. Secondary traumatization is the process via an indirect exposure, which may develop from hearing about a traumatic event or caring for someone who has experienced a traumatic event. This process may lead to burnout, compassion fatigue, vicarious trauma, and secondary traumatic stress [20,22].

### Burnout

Burnout (BO), an emotional and behavioral impairment that results from the exposure to high levels of occupational stress, has been described as a combination of three factors: emotional exhaustion, depersonalization and personal accomplishment [23]. Individuals who are at risk of a BO, usually have some level of perfectionism and feel guilty if they do not perform as well as they would like to. This goal-oriented mindset could cause an extreme imbalance in work-related situations and might lead to long-term absenteeism. Although BO can be severe, it has also been viewed as a contagious syndrome [24]. The social context, and especially the interaction with complaining colleagues, might play an important role in the development of BO. Furthermore, BO has been mentioned as a fashionable diagnosis because a clear and standardized definition is lacking [8,25]. A substantial number of studies on BO in a broad range of professions were published and a peak in media coverage occurred since the first description [26]. However, since its origination, the operationalization and measurement of BO have differed enormously.

### Compassion Fatigue

Compassion fatigue (CF) has been defined as a state of physical or psychological distress in caregivers, which occurs as a consequence of an ongoing and snowballing process in a demanding relationship with needy individuals [27,28]. It has been associated with a ‘helper syndrome’ that results from continuous disappointing situations and leads to moral distress [29]. CF was described for the first time in the early nineties as the loss of compassion in result of repeated exposure to suffering during work [30]. A little later, CF was defined as secondary traumatic stress (STS) resulting from a deep involvement with a primarily traumatized person, because of the “more friendly framing” [29]. From this time on, CF has interchangeably been referred to as secondary- and posttraumatic stress (S/PTS) or vicarious trauma (VT) [27–29,31]. CF consists of two parts. The first part contains issues such as exhaustion, frustration, and depression, typical associated with BO. The second part is the negative feeling driven by concerns such as hyper-vigilance, avoidance, fear and intrusion, which are also characteristics of S/PTS.

### Relationships of Concepts

Although BO is closely related to CF, the underlying mechanism most likely differs. BO is believed to be related to occupational factors, such as workload, autonomy, and rewarding, rather than personal relationships [32]. In contrast, an inability to engage, or enter into a caring relationship, is considered to be the core of CF [33]. What becomes more and more apparent is the level of complexity in the various concepts and mutual relationships. Besides the already mentioned interchangeably usage of CF and STS, a significant positive correlation between CF and BO was found in some studies, suggesting an overlay in one or more of the components of these phenomena [20,22]. According to Elkonin and Lizelle, BO illustrates the end result of traumatic stress in the professional life of the caregiver and could be an extreme case of CF [22]. Conversely, Sabo suggested BO as a pre-condition for CF [33], and Aycock proposed that CF replaces the outdated notion of BO in describing the phenomenon in oncology nurses [34]. This review explores all mentioned concepts, taken together in this study as emotional distress, because of the same range of causes, coping mechanisms, and consequences in the field of traumatization.

### Aim of the Study

The main purpose of this review was to evaluate the literature on emotional distress among professionals in the ICU according the PRISMA method, with an emphasis on the prevalence of burnout and compassion fatigue. We enhanced some new knowledge in this field to assess the current literature precisely and compare the measuring instruments and the results of the studies. Furthermore, while the sometimes devastating personal and organizational consequences of BO and CF have been published previously, very few studies have addressed the effectiveness of preventive strategies. This review aims to provide a starting point for clinical practice guideline developers and summarizes interventions to prevent the negative consequences of emotional distress among healthcare professionals in the ICU. The following research questions have been addressed:

1. What is the prevalence of compassion fatigue and burnout among healthcare professionals in the ICU?
2. Which preventive strategies have been successfully applied to reduce emotional distress among ICU professionals?

## Methods

A systematic review of the scientific literature was conducted to obtain original articles for appraisal. Pre-determined search strategies were followed and quality criteria were applied as guidelines to conduct the review process [35]. The current study was performed in accordance with the PRISMA statement (S1 PRISMA 2009 Checklist) [36]. This review study did not need ethical approval nor was individual consent needed.

### Search Strategy

A systematic search in the computerized databases of Embase, Medline OvidSP, Cinahl, Web-of-science, PsychINFO, PubMed publisher, Cochrane and Google Scholar has been performed. The following Medical Subject Headings (MeSH) were used: burnout, empathy and fatigue. This search was supplemented with compassion fatigue and secondary traumatic stress as free text words. The Boolean indicator ‘AND’ was used to select the studies applied to the ICU healthcare professionals. All terms were tailored to the thesaurus of each database, the complete search strategy is recorded in the protocol (S1 Table). Local unpublished surveys, unpublished reports and academic theses were not included. All references were retrieved, organized and stored with EndNote X7.1 version 17.

### Eligibility Criteria

In the first round, the references from each database were screened by the title and abstract for relevancy. We included studies that 1) dealt with the prevalence, as described in the article or calculated from the presented data, or 2) described an intervention on BO, CF, VT or S/PTS. All studies were set within an ICU, Critical Care Unit, Neonatology Intensive Care Unit or Pediatric Intensive Care Unit, and were applicable to healthcare professionals i.e. nurses or physicians. We were particularly interested in effects of the interventions on the professional quality of life of the individual workers. We chose 1992 as the initial search year because the first article on CF in nurses was published that year [30], the search included original articles written in the English language all years through 30 June 2014. We excluded studies on coping with work stress and the causes and consequences of BO.

After the full text was read in the second selection round, the articles were limited to the prevalence presented as percentages or numbers of BO, CF, VT or S/PTS and intervention studies in which respondents are being pre- and post-tested or compared in two groups in different regimes. Finally, the included articles were manually checked for new references until no further studies were identified.

### Qualitative Data Extraction

A set of quality criteria was developed to assess the methodological soundness [27,35], see Table 1. The total study quality has been computed as 12.5% for each positive scored criterion, at least six of eight criteria should be applicable.

**Table 1:** Set of criteria used to appraise the study quality.

	Quality criterion	Yes/No
1	Clear research questions and objectives	
2	A definition of the measured concept(s)	
3	Valid and reliable measuring instrument(s)	
4	Method description in detail	
5	Information on the size and type of the target population	
6	Information on the number and characteristics of the subjects who agreed to participate	
7	Addressing missing values	
8	Appropriate statistical analysis	

Three of the authors (MvM, MN and EK) independently extracted qualitative information from each article. The following information was determined: ´bibliographic information´ (e.g., first author, year of publication), ´aim of the study´, ´definition of concepts´, ´setting´ (e.g., general or academic hospital), ´population and sample size´ (e.g., nurses or physicians), ´method design´, ´measuring instrument, validation and reliability´, and ´prevalence´. Disagreements between the three reviewers were discussed until a consensus was reached.

## Results

**Fig 1:**
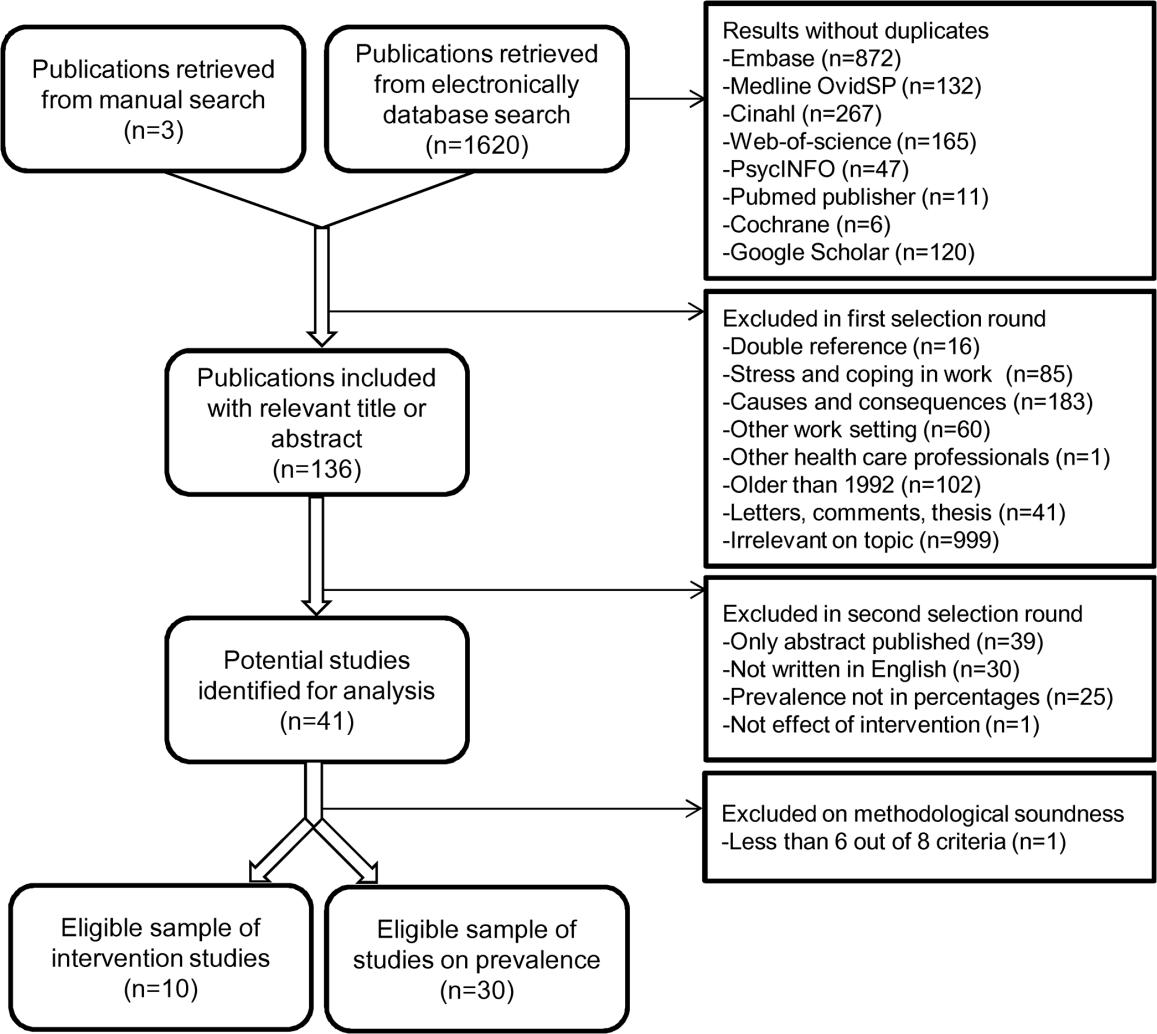
Flowchart review process. An adapted PRISMA flowchart of the total review process on the prevalence of compassion fatigue and burnout among healthcare professionals in the intensive care unit.

The review process, which is illustrated in Fig 1, began with 2580 references retrieved from the electronic databases. Deleting duplicate references (n = 1620) and a manual search (n = 3) resulted in 136 relevant publications after the first selection round. Subsequently, the references only published as an abstract (n = 39) or non English (n = 30) were removed. A few studies were excluded because prevalence could not be calculated from the presented data [37–39] or effects of the intervention were not measured [17]. Finally, a sample of 30 eligible articles on the prevalence of emotional distress and 10 associated intervention studies were appraised as methodologically sound and included for extensive review [20,22,40–77]. The assessment of all articles which were read in full text, as indicated in additional file S2, had an excellent inter-rater agreement using Cohen´s kappa (*k* = 0.912).

An overview of the included publications, with the study characteristics such as setting, sample size, sample characteristics and quality assessment, is provided in Table 2. Most of the studies were conducted in Europe and North-America (70%), nine studies were conducted in an academic or tertiary hospital setting (22.5%) and other study settings included small, large or a mix of hospital samples. The number of respondents varied between 25 and 3,052; in most studies female respondents were over-represented. The response rates varied between 24.8% [50] and 98.8% [77]. In Table 3 all the sample characteristics are summarized.

**Table 2:** An overview of the included articles with the study characteristics.

First author Year of publication	Setting	Sample size (response rate)	Sample characteristics	Design	Study quality (%)*
Ali, et al., 2011	5 ICUs in the United States	39 86.7%	Intensivist. Female 24%; Mean age 41 years. Comparison of a continuous staffing schedule in half month rotation, and an interrupted schedule with weekend cross-coverage by colleagues, on the level of burnout.	Prospective, cluster-randomized, alternating trial	75
Barbosa, et al., 2012	6 ICUs in Brazil	76	Physicians. Female 55.22%; Mean age 43.9 years; mean length of time since graduation 19.4 years	Cross-sectional observational descriptive study	87.5
Bellieni, et al., 2012	NICUs in Italy	110 (84.6%)	Neonatologist. Female 60.0%; Age 35–50 (78.2%); Years of service <5 (30%), 5–10 (33.6%), >15 (36.4%)	Cross-sectional study	100
Cho, et al., 2009	65 ICUs in Korea	1365 (93%)	Charge and staff Nurses. Female 98.9%; Plan to leave 26.3%	Cross-sectional study	100
Czaja, et al., 2012	A tertiary-care PICU in the USA	173 (43%)	Nurses. Female 93.0%; Mean age 35 years; Considering change in careers 31%	Cross-sectional study	100
Eagle, et al 2012	PICU in New Orleans	T_1_ 28 and T_2_ 22	Physicians and nurses. Work experience modus 1–5 years (57%). Measuring the effect of an educational session and skills training of coping, grief and peer support on the level of burnout.	Pre- post design	87.5
Elkonin, et al., 2011	Three ICUs in Africa	30 (40%)	Nurses. Female 93.33%; Mean age 38.7 years; Years of ICU experience 1–10 (63%), 11–20 (23.33%)	Quantitative exploratory descriptive study	87.5
Embriaco, et al., 2007	189 ICUs in France	978 (82.3%)	Physicians, fellows, interns or residents Female 28%; Mean age 40 years; Mean working hours per week 59 ± 12	Observational survey study	100
Galvan, et al.2012	All PICUs of Argentina	162 (60%)	Physicians with a workload equal or more than 24 hours/week. Female 57%; Mean age 42 years; No plans to continue with PICU activity 31%	Observational cross-sectional study	100
Garland, et al., 2012	2 ICUs in the United States	37 (94.9%)	Intensivist. Female 8%; Age 41–60; ICU training > 10 years ago 56%. Comparison of a standard model, where one intensivist worked for 7 days, taking night call from home, and the shift work model, where one intensivist worked 7 day shifts, while other intensivist remained in the ICU at night, on the level of burnout.	Alternating cross-over design	87.5
Goetz, et al., 2012	1 ICU in Germany	86 (54.4%)	Nurses; ICU (n = 57) and IMC (n = 29). Female 76.7%; Age between 25 and 34 years (56.1%)	Cross-sectional study	100
Guntupalli, et al., 1996	ICUs in the USA	248 (24.8%)	Physicians. Female 11.3%; Mean age 41.6 years, 47.3% indicated they would wind down critical care component in 9.9 ± 4.7 years	Cross-sectional study	87.5
Guntupalli, et al., 2014	ICU in Houston	213	Nurses (n = 151) and Respiratory therapist (n = 62). Female 72.2%	Cross-sectional study	75
Karanikola, et al., 2012	ICUs in 8 general hospitals in Greece.	152 (60.0%)	Nurses. Female 78.8%; Mean age 31.8 years, Mean work experience in ICU 5.0 years	Descriptive correlational design	100
Lederer, et al. 2008	Five ICUs in Austria.	183 (59%)	Nurses (n = 150) and physicians (n = 33). Female 56.8%; Age 20–29 (28.4%), 30–39 (55.7%), >40 (15.8%), Years of employment <1–5 (41.0%), 5–10 (28.4%), >10 (30.6%)	Prospective cross-sectional study	100
Liu, K., et al., 2012	Adult general, specialty medical, surgical and ICU in China.	1104 (95.5%)	Nurses. emale 97.8%; Mean age 28.55 years; Years of employment <5 (41.0%), 5–10 (28.4%), >10 (30.6%)	Cross-sectional study	87,5
Liu,Y., et al., 2013	12 CCUs in Shanghai.	215 (97.7%)	Nurses. Female 98.6%; Age <30 (62.3%), 30–39 (34%), >40 (3.7%); Years of CCU experience <5 (57.2%), 5–9 (23.3%), >10 (19.6%)	Cross-sectional study	87.5
Loiselle, et al., 2011	ICU in Canada	T_1_ 44 and T_2_ 27 (45% and 28%)	Nurses. Female 91.9%; Age modus 25–34 (62.2%) Measuring the effect of the Adler/Sheiner Programme (structural elements on information and support in Family Centered Care) on emotional distress.	Pre-experimental mixed design using quantitative and qualitative methods	87.5
Mason, et al., 2014	ICU in the United States	26 (77%)	Nurses. Modus 21–30 years ICU experience (61.5%)	Non-experimental, descriptive, correlational design	87.5
Meadors, et al., 2008	PICU, NICU and PEDS in the United States	185	Nurses and other (e.g social worker). Female 96.8%; Mean age 35.3 years; Mean current position 7.4 years Measuring the effect of a 4 hours educational seminar dealing with compassion fatigue, management of stress and factors associates with grief, on personal stress.	Pre- post design	87.5
Meadors, et al., 2010	All PICUs and NICUs in the United States	167	Nurses (23), Physicians (21), Chaplains (22), Child life specialist (87) and other (8). Female 82.0%; Average working experience in their unit 6–10 years	A correlational design	100
Mealer, et al., 2007	3 ICUs and 3 general wards in America	351 (47%)	Full-time nurses ICU (n = 230) and general ward (n = 121). Female 86.6; Mean age 37.6 years	Cross-sectional study	87.5
Mehrabi, et al., 2012	ICU in Iran	34 94.4%	Nurses. Female 100%; Mean age 33.5 years Measuring the effect of an 8 weeks yoga class on stress coping strategies.	Quasi experimental pre- post design	75
Merlani, et al., 2011	74 of the 92 certified ICUs in Switzerland.	3052 (71%)	Nurses (n = 2587) and physicians (n = 465). Female76%; Age <40 years (70%); Mean years of ICU experience 7.5	Prospective, multicenter, observational survey	100
Nooryan, et al 2011	ICU, CCU, PICU, psychiatry and burn wards in Armenia	106 70.7%	Physicians and nurses. Mean age case group 33.2 and control group 31.6 years; Measuring the effect of a training programme dealing with emotional intelligence on situational and personality anxiety.	Cross interventional, pre-and post, case and control group design	87.5
Nooryan, et al 2012	ICU, CCU, PICU, psychiatry and burn wards in Iran	150	Physicians and nurses. Mean age case group 38.8 and control group 39.7 years; Average work experience nurses 7.4 and physicians 4.4 years. Measuring the effect of a training programme dealing with emotional intelligence on situational and personality anxiety.	Cross interventional, pre-and post, case and control group design	87.5
Poncet, et al., 2007	165 ICUs in France	2392 (95.8%)	Nurses. Female 82.1%, Mean age 31 years, Mean months in ICU 40 (17 to 96)	Cross-sectional study	87.5
Quenot, et al., 2012	1 ICU in France	Period 1 n = 53 (85%) and period 2 n = 49 (79%)	Nurses (49 and 45) and physicians (4 and 4). Female 40% and 36% in respective, Mean age 27 and 26 years in respective, ICU experience > 5 years 63% and 65% in respective	Longitudinal, monocentric, before-and-after interventional study	100
Raftopoulos, et al., 2012	ICU, general and emergency department in Cyprus	1482 (98.6%)	Nurses during the provision of a training program for upgrading from diploma to bachelor level. Female 80.8%, Mean age 36.68 years, Mean working experience 14.53 years	Cross-sectional study	100
Raggio, et al., 2007	Two ICUs in Italy	50	Nurses (n = 25) and physicians (n = 25). Female 40.0% Mean age men 42.2 and woman 38.1 years	Observational study by administration of psychometric test	100
Rochefort, et al., 2010	9 NICUs Canada	339 (61.3%)	Registered nurses. Female 98.5%, Mean age 39.4 years; Mean of NICU experience 12.4 years	Cross-sectional study	100
Saini, et al., 2011		25	Nurses. Female 92.0%, Mean age 27.9 years; Mean of ICU experience 3.2 years	Cross-sectional mixed method design	75
Shehabi, et al., 2008	Australian ICUs	115 (36%)	Intensivists. No demographic data	Cross-sectional study	87.5
Sluiter, et al., 2005	PICU in the Netherlands	50 and 36 55% overall	Physicians and nurses. Mean age 41 years; Mean of PICU experience 11 years. Measuring the effect of a structured multidisciplinary work shift evaluation on the level of burnout.	Prospective, repeated measurements design	87.5
Su, et al., 2007	The Veterans General Hospital in Taipei City, China	102	Nurses SARS regular (n = 44), SARS ICU (n = 26), neurology (n = 15) and CCU (n = 17). Female 100%; Mean age 29.8, 31.5, 25.4 and 32.7 years in respective	A prospective and periodic follow-up design study	87.5
Teixeira, et al., 2013	10 ICUs Portugal	300 (67%)	Physicians (n = 82) and nurses (n = 218). Female 65.0%; Mean age 32 years	Cross-sectional study	100
Verdon, et al., 2008	ICU in Switzerland	97 (91%)	Nurses (n = 86) and nurse-assistants (n = 11). Female 61%	Cross-sectional study	100
West, et al.,	ICU in the United States	74 study and 350 non-study 75% overall	Physicians. Female 33.8%. Measuring the effect of a 19 biweekly facilitated discussion groups incorporating elements of mindfulness, reflection, shared experience and small-group learning on the level of burnout.	Randomized clinical trial testing an intervention	100
Young, et al., 2011	CCU and IMC in the USA	70	Nurses ICU (n = 45) and IMC (n = 25). No demographic data	Exploratory descriptive study	75
Zhang, et al, 2014	14 ICUs in Liaoning, China	426 (98.8%)	Nurses. Female 88.5%; Median age 25 years	Cross-sectional observational study	100

ICU = Intensive care Unit, IMC = Inter Mediate Care, CCU = Corony Care Unit, PICU = Pediatric Intensive Care Unit, NICU = Neonatal Intensive Care Unit

* Study Quality is computed as 12.5% for each positive scored quality criterion (see Table 1), at least six of eight criteria should be applicable

**Table 3:** The sample characteristics of the included studies.

Variable		Number (percentage)
Continent		
	Europe	14 (35.0%)
	North-America	14 (35.0%)
	Asia	8 (20.0%)
	South-America	2 (5.0%)
	Australia	1 (2.5%)
	Africa	1 (2.5%)
Hospital setting		
	Academic or tertiary hospital setting	9 (22.5%)
	Other	31 (77.5%)
Specialism		
	Intensive care unit	25 (62.5%)
	Corony care unit	2 (5.0%)
	Neonatology intensive care unit and pediatric intensive care unit	8 (20.0%)
	Comparison of different wards	5 (12.5%)
Occupation		
	Nursing profession	20 (50.0%)
	Medical profession	8 (20.0%)
	Mix of nurses and physicians	11 (27.5%)
	Other	1 (2.5%)
Range in number of respondents		25 to 3,052
Total respondents		14,770
Response rate		24.8% to 98.8%
Range in percentage of female respondents		8.0% to 100.0%

### Prevalence

Studies on the prevalence of CF and S/PTS in the ICU were less frequent than studies of BO, as shown in Table 4, and only one study mentioned VT [22]. The Professional Quality of Care (ProQOL) questionnaire, which was used in some of the reviewed studies, was developed to measure both CF and BO [78]. Additionally, this questionnaire distinguishes also the positive effects of caring, referred to as compassion satisfaction. Over time, this tool has been validated in various healthcare work environments and has proven to be reliable and feasible for medical staff [27,79]. According to the ProQOL-revisited V, two different studies showed 7.3% [20] and 40% [22] of the respondents who scored high on CF compared with 1.2% and 23%, respectively, who had severe BO. Two other studies, which were using the ProQOL, measured a 0% high risk for BO, as well as S/PTS as stated by the authors [57,76]. However, succeeding studies described the prevalence of S/PTS as 17%, using the Posttraumatic Diagnostic Scale [44], and 38.5% using the Davidson Trauma Scale [72]. Additionally, 24% of 230 full-time working ICU nurses in a university hospital experienced some symptoms of S/PTS, such as nightmares, according to results from the Post Traumatic Stress Syndrome 10 Questions Inventory, compared to 14% in general nurses and 29% in the control group [60].

**Table 4:** Amount of articles on the prevalence of emotional distress and prevalence range.

		Mentioned in study (n)	Prevalence range (%)
Burnout		28 (93.3%)	0.0–70.1
	Emotional exhaustion		7.6–52.0
	Depersonalization		3.3–41.8
	Personal accomplishment		6.0–75.9
Compassion fatigue		5 (16.7%)	7.3–40.0
Secondary- and post-traumatic stress		6 (20.0%)	0.0–38.5
Vicarious trauma or stress		1 (3.3%)	

BO is mostly assessed by the Maslach Burnout Inventory (MBI), according to some authors as the standard tool for measuring the severity of BO [2,41,66]. The MBI is a highly reliable and validated 22-item self-report questionnaire that evaluates the three domains of BO in independent subscales: emotional exhaustion, depersonalization and personal accomplishment. The MBI was predominately used (n = 22, 70.0%), including the French (n = 4), the Portuguese (n = 2), the Chinese (n = 2), the Korean (n = 1) and the German (n = 1) validated versions, in addition to the original English version.

The reported prevalence rate of BO in the ICU, measured with the MBI, varied from 14.0%, after a preventive intervention [65], to 70.1% when BO was defined as a high score on only one subscale [41]. The latter study also stated that the prevalence would be 17.7% if BO had been defined as a high score on the combined subscales. Similarly, Czaja *et al*. reported a prevalence rate of 68.0% with BO defined as a high score on any BO symptom, and 45% for the emotional exhaustion subscale [44]. Some other studies defined a high risk for BO by a cut-off score in the emotional exhaustion subscale, leading to estimates of prevalence varying from 25.0% to 51.9% [50,51,55,67,70]; four studies defined BO by a total MBI score of > -9 and reported the prevalence in the range of 28.0% to 46.5% [46,61,64,74].

One study presented a significantly lower prevalence of BO in ICU healthcare professionals (n = 121); 14.5% in the ICU compared to 21.9% in the oncology department (n = 82), 17.5% in the operating theatre (n = 88), 17.2% in the surgical department (n = 134), and 12.4% in the medical department (n = 109) [66]. No difference for the Neonatology Intensive Care Unit or Pediatric Intensive Care Unit, with the prevalence ranging from 1.2% [20] to 41% [47], was found compared to the adult ICU, with the prevalence ranging from 16% [77] to 46.5% [46], measured with the MBI. Correspondingly, no clustering of prevalence rates was identified for specific hospital settings (i.e., an academic or regional hospital), professional role (i.e., doctors or nurses), or number of respondents in the study group.

A summary of the diverse measurement instruments, cut-off scores and reported prevalence, are shown in Table 5.

**Table 5:** A summary of the diverse measuring instruments, cut-off scores and found prevalences.

Concept*	Measuring instrument		Applied in	Prevalence of high risk
CF	Professional Quality of Care–Revision IV, CF subscale *CF > 17 high*, *8–17 average and <8 low risk*		Elkonin 2011	40.0%
			Meadors 2010	7.3%
S/PTS	Professional Quality of Care–Revision V, CF subscale *BO >56 high*, *55–43 moderate and < 42 low risk*		Young 2011	0.0%
			Mason 2014	0.0%
	Posttraumatic Diagnostic Scale		Czaja 2012	17.0%
	Post Traumatic Stress Syndrome 10 Questions Inventory		Mealer 2007	24.0%
	Davidson Trauma Scale		Su 2007	38.5%
BO	Professional Quality of Care–Revision IV, BO subscale *BO >27 high*, *18–27 moderate and < 18 low risk*		Elkonin 2011	23.0%
			Meadors 2010	1.2%
	Professional Quality of Care–Revision V, BO subscale *BO >56 high*, *55–44 moderate and < 43 low risk*		Young 2011	0.0%
			Mason 2014	0.0%
	Maslach Burnout Inventory with three subscales; EE**(9 items), DP**(5 items) and PA**(8 items)			
		A high score on EE subscale. *EE ≥ 27 high*, *19–26 moderate and ≤ 19 low score*	Cho 2009	53.0%
			Liu 2012	37.3%
		A high score on EE, *cut-off score not defined*	Rochefort 2010	35.7%
		A high score in one subscale. *EE ≥ 27 high*, *19–26 moderate and ≤ 19 low score*, *DP ≥ 12 high*, *6–11 moderate and < 6 low score*, *PA 0–33 high*, *34–39 moderate*, *and ≥ 40 low score*	Barbosa 2012	70.1%
			Galvan 2012	41.0%
		A high score in one subscale *EE > 24*, *DP > 9 or PA < 29*	Raggio2007	EE 32.0%
		A high score in one subscale *EE ≥ 27*, *DP ≥ 10 or PA ≤ 33*	Liu 2013	EE 51.9%
		A high score in one subscale or a total score > -9 *EE > 30*, *DP >12 or PA < 33*	Quenot 2012	28.0% before14.0% after
		A high score on EE and DP *EE ≥ 30 high*, *18–29 moderate and ≤ 17 low score*, *DP ≥ 10 high*, *6–9 moderate and ≤ 6 low score*, *PA 0–33 high*, *34–39 moderate*, *and ≥ 40 low score*	Raftopoulos 2012	14.5%
		A high score in two of the three subscales *EE ≥ 25 high*, *15–24 moderate and ≤ 14 low score*, *DP ≥ 190 high*, *4–9 moderate and ≤ 3 low score*, *PA 0–32 high*, *33–39 moderate*, *and ≥ 40 low score*	Teixeira 2013	31.0%
		A high score on EE and DP with a low score on PA subscales *EE ≥ 27 high*, *17–26 moderate and ≤ 16 low score*, *DP ≥ 14 high*, *9–13 moderate and ≤ 8 low score*, *PA 0–30 high*, *31–36 moderate*, *and ≥ 37 low score*	Guntupalli 2014	EE 25.0%
		A high score on EE and DP with a low score on PA subscales *EE ≥ 30 high*, *18–29 moderate and ≤ 17 low score*, *DP ≥ 10 high*, *6–9 moderate and < 6 low score*, *PA 0–33 high*, *34–39 moderate*, *and ≥ 40 low score*	Karanikola 2012	25.0%
		A high score on EE and DP with a low score on PA subscales *EE > 31 high*, *21–30 moderate and <20 low score*, *DP > 11 high*, *6–10 moderate and < 5 low score*, *PA 0–35 high*, *36–41 moderate*, *and > 42 low score*	Guntupalli 1996	EE 29.0%
			Zhang 2014	16.0%
		A moderate to high score one subscale *EE ≥ 17*, *DP ≥ 7 and PA ≤ 39*	Czaja	68.0%
		A total MBI score > -9	Embriaco 2007	46.5%
			Merlani 2011	28.0%
			Poncet 2007	32.8%
			Verdon 2008	28.0%
		High level not defined	Shehabi 2008	EE 42.0%
	Maslach Burnout Inventory, with four subscales; EE (9 items), DP (5 items), PA (7 items) and consternation (4 items)		Lederer 2008	34.4%
	Link Burnout Questionnaire		Bellieni 2012	30.0%
	The Arbeisbezogene Verhaltens- und Erlebensmuster (Burnout pattern)		Goetz 2012	17.7%

* CF = Compassion fatigue, S/PTS = Secondary- and post-traumatic stress, BO = Burnout

**EE = Emotional exhaustion, DP = Depersonalization, PA = Personal accomplishment

The included studies reported a broad range of variables related to emotional distress, see Table 6. Work environment [22,46,68,74], professional role [61,67] and conflicts [46,64] were significantly and positively related to the measured phenomenon. However, some studies stated opposite results. Most confusing variable was the female sex, with an increasing [46,66] versus a decreasing [61] effect, and no significantly measured influence [42,50,52,64] on emotional distress.

**Table 6:** Relationship of a variable with emotional distress, pro and con.

Variable	Pro: significantly related to emotional distress	Con: significantly not related to emotional distress
High workload	Embriaco 2007	Barbosa 2012
	Poncet 2007	
Short work experience	Bellieni 2012	Karakinola 2012
	Liu 2012	
	Zhang 2014	
Work environment	Elkonin 2011	
	Embriaco 2007	
	Verdon 2008	
	Rochefort 2010	
Nurse/patient ratio	Cho 2009	
Professional role (nurse-doctor)	Raggio 2007 (nurse)	
	Merlani 2001 (nurse ass)	
End-of-life care	Poncet 2007	Czaja 2012
Mortality rate	Merlanie 2011	Embriaco 2007
Demographic variables	Poncet 2007 (age)	Czaja 2012
	Raftopoulos 2012 (age)	Karakinola 2012
	Bellieni 2012 (age)	Lederer 2008
	Merlanie 2011 (age)	Guntupalli 1996 (age)
	Liu 2012 (age)	Guntupalli 2014 (age
Having children	Bellieni 2012	
Female sex	Embriaco 2007 (increased)	Poncet 2007
	Raftopoulos 2012 (increased)	Bellieni 2012
	Raggio 2007 (increased EE)	Guntupalli 1996
	Merlani 2011 (decreased)	Karakinola 2012
		Guntupalli 2014
Conflicts	Embriaco 2007	
	Poncet 2007	
Number of ICU beds		Guntupalli 1996

### Preventive Strategies

A wide range of intervention strategies to reduce emotional distress among ICU professionals emerge from the recent literature, see Table 7. Ten studies measured the effect of an intervention, such as different intensivist work schedules [40,48], educational programs on emotional distress [45,58], improving elements of family-centered care and communication skills [56,65,71], strategies regarding personality and coping [62,63], and relaxation exercises [59,75] such as yoga and mindfulness. In addition, seven of the included studies suggested preventive strategies, varying from improving the work environment [49,55,68], focussing more on social support and individual coping strategies [54], changing team composition to include a greater number of women [61], developing teambuilding and periodic job rotation [42], and a mix of all these elements [67].

**Table 7:** Summary of the interventions on emotional distress.

Type of intervention		Description of intervention	Study
Organization-directed interventions			
		Work schedules of intensivist	Ali *et al*. 2011
			Garland *et al*. 2012
		Improving work environment	Goets *et al*. 2012
			Liu *et al*. 2013
			Rochefort *et al*. 2010
		Change team composition	Merlani *et al*. 2011
		Teambuilding and job rotation	Bellieni *et al*. 2012
Person-directed interventions			
	Practical		
		Educational programs, seminars	Eagle *et al*. 2012
			Meadors, *et al* 2008
			West *et al*. 2014
		Improve communication skills	Loiselle *et al*. 2012
			Quenot *et al*. 2012
			Sluiter *et al*. 2005
		Relaxation exercises	West *et al*. 2014
			Mehrabi *et al*. 2012
		Mindfulness	West *et al*. 2014
	Personal		
		Personality and coping	Nooryan *et al*. 2011
			Nooryan *et al*. 2012
		Social support and individual coping	Liu *et al*. 2012
		Counselling	Lederer *et al*. 2008

According to Quenot *et al*. [65], the implementation of a set of active, intensive communication strategies regarding end-of-life care in the ICU has been associated with significantly lower rates of BO after the intervention. These strategies comprised elements in the organization, (i.e., the introduction of unrestricted visiting hours and the availability of a staff psychologist for consultation on demand), communication, (i.e., daily meetings of the caregiving team with the patient and/or their family and the discussion of palliative care options), ethics, (i.e., a special section in every patient´s medical record or ethical rounds), and stress debriefings and conflict prevention. Reductions of almost 50% and 60% were reported in the relative risk of BO and depression, respectively, after some of these interventions. Another promising preventive strategy is mindfulness training. West *et al*. [80] measured a positive effect of 19 biweekly discussion groups, which included elements of mindfulness, reflection and shared experience, on physician well-being. Furthermore, Lederer *et al*. [53] mentioned a positive influence on the prevalence of a fully developed BO due to the support of a facilitator. An external psychologist provided support whenever needed in two of the five ICUs included in this study; more specifically, individuals with a high risk of BO were less likely to consult the psychologist. In contrast, peer support had no significant effect on BO [45]. Finally, educational seminars on CF increased both awareness and resources for the prevention of emotional distress in the future [58]. The participants in that study felt significantly less tense and reported being more calm and peaceful after the intervention.

## Discussion

This comprehensive systematic review identified thirty studies that investigated the prevalence of BO, CF, VT or S/PTS among healthcare professionals working in ICUs. It is clear that working at an ICU correlates with a substantial risk of emotional distress, all of the included studies underscored the stressful environment in the ICU. From this perspective, it is even more strikingly to find contradictory results with lower percentages or means on BO, CF or S/PTS in the ICU compared to other wards [20,44,66,72], which is also esthablished in supplementary studies [79,81]. This anomaly might be explained through unique personal qualities, such as resilience, empathic ability, coping mechanisms or emotional intelligence, and environmental factors, such as training, mental support, organizational culture or the differences between cultures and countries.

Although the risk of emotional distress has been recognized in this review, the true magnitude of the explored phenomena remains unclear for several reasons. First, the definitions of the types of distress have been used interchangeably across studies; more specifically, CF has been measured with the same subscale of the ProQOL as S/PTS [20,22,76]. One of the key elements in the ProQOL model is the empathic ability of the caregivers and the therapeutic relationship with clients. However, a profound analysis has shown that STS and CF really differ regarding their content validity [82]. In contrast to STS, which refers to symptoms related to a process of indirect traumatization, CF is stressing the diminished sympathy to someone´s suffering, and the lessened desire to help in a broad context through the meaning of compassion [20,83]. The loss of this compassionate energy is also mentioned in a conceptual analysis of CF, in which is stated that the synonymous use of CF with STS is far removed from Joinson´s original meaning [28]. In addition, Sabo suggested that the binary dimension of CF in the ProQOL (i.e., you either have it or not), is not congruent human nature, which is characterized by gradual responses similar to slightly, moderately or severely. More fundamentally, the model also failed to clearly conceptualize empathy, thus making it difficult to understand the background of CF [33]. Therefore, Coetzee and Klopper [28]^p237^ distinguished CF again as a loss of the nurturing ability that is vital to compassionate care. The essential issue of the caring professionals is to deliver themselves; being present and empathic. If this process stagnates, the emotional price of caring can become a burden in personal life, manifested by emotional distress such as CF.

The Secondary Traumatic Stress Scale is the only instrument that is designed to assess the symptoms of STS by a 17-item Likert scale [18], however, this questionnaire has not been used among ICU professionals. The ProQOL has been used in many healthcare settings, is profoundly tested, and marked as a valid and reliable instrument [84]. However, the STS/CF subscale is fundamentally based on the concept of STS, with items explicitly pointing at traumatic stress reactions such as a startle reflex, intrusive and frightening thoughts, re-experiencing situations, and avoidance. To sum up, there is no measurement instrument to assess CF in the meaning of a lost ability to care.

Second, the reported prevalence of emotional distress differed based on the applied measurement instruments. The ProQOL seemed incapable of detecting a risk for severe burnout, which is illustrated with prevalence rates around zero [20,57,76], in comparison, the prevalence with the MBI ranged from 14.0% up to 70.1%. Meadors *et al*. provided a valuable and comprehensive overview of the mental trauma literature in the non-adult ICUs, and found a low prevalence of CF (7.3%) and BO (1.2%) with the ProQOL [20]. They suggested that drop out by the already over-exhausted individuals participating in the study to explain their results; this reason of self-selection bias may have a substantial role in all of the studies on this topic. In contrast to these results, another study among 162 intensivists working at a paediatric intensive care in Argentina described a 41% BO prevalence measured by the MBI [47], and a study of 173 nurses working at a tertiary children´s hospital found that 68% of respondents had at least one BO symptom [44]. In short, the MBI is characterized by a more discriminative power than the ProQOL. Nevertheless, the highest prevalence of CF, defined by the authors as such and measured by the ProQOL, was 40.0% and reported in a study of 30 registered nurses in two ICUs in South Africa [22]; a 23% prevalence of BO was found in the same study. It was stated that there was a noteworthy shortage of ICU nurses in that country, and most of the participants were not trained for nursing critically ill patients. Thus, the work environment might have been particularly stressful due to a lack of appropriate nursing skills and ICU knowledge. The highest prevalence of S/PTS, 38.5%, was measured with the Davidson Trauma Scale in a study of 26 ICU-nurses in a SARS unit in Taiwan; this group was compared to 17 critical care and 15 neurology nurses working in two non-SARS units [72]. However, a study using the ProQOL in an academic hospital with 68 nurses in the United States did not find a severe risk for S/PTS [76]. This difference might be explained partially by the extreme working conditions associated with the SARS outbreak and the difference in measuring instruments. Although the last two studies addressed relevant issues, it may be difficult to identify changeable determinants in the work setting to prevent the consequences of emotional distress.

Third, the outcome scales or cut-off points used to indicate the prevalence of burnout measured with the MBI have a wide range, as presented in Table 5. A great deal of work has been done with the MBI, both conceptualizing and measuring BO in a valid and reliable way [85]. However, it should be used uniformly, with an evaluation of all three subscales together. As shown, Czaja *et al*. [44] used a moderate to high score in one subscale with emotional exhaustion above 17 to establish the prevalence of BO, with 68% as a result. In contrast, Zhang *et al*.[77] found a prevalence of 16%, with BO defined a high score on emotional exhaustion (above 31), depersonalization (above 11), and a low score on personal accomplishment (above 42). Schaufeli and Van Dierendonk (1995) stated that caution is needed when cut-off points are used to classify the severity of BO, which could also be nation specific [86]. All in all, the prevalence might be affected by the used measuring instrument as well as the different cut-off points and subscales. Finally, the variety of research variables, i.e., whether the variables are significantly related to emotional distress in the ICU, perpetuates the lack of clarity.

Therefore, the true prevalence of BO, CF, S/PTS and VT in ICU-professionals remains open for discussion, which might emphasize the need for a ´gold standard´ which will be used in all future research. To begin, the concepts specifically related to the ICU healthcare environment have to be defined by a wide-ranging consensus committee, e.g by conducting a Delphi study. Subsequently, more agreement is needed to address the discrepancies in measurement issues, and to better investigate emotional distress with a large international quantitative observational multicenter study. Only one such study has been published to date [24], from which an impressive amount of data on burnout were already gathered in 1994. The results were reported not sooner than 2005 because they formed part of a larger study on the organizational influence on the effectiveness and efficiency of ICUs. This study is still of importance because of the focus on fundamental psychological processes, such as emotional contagion in burnout, and the relationships between variables. However, the prevalence of BO among ICU professionals might change over time and a broader view on emotional distress would be preferable.

It is highly recommended to further investigate and compare the consequences of emotional distress in the ICU in a valid comparative manner to indicate the relevance of the problem. However, cross-sectional study designs cannot reveal causal relationships between contributing variables, individual coping mechanisms or organizational preventive strategies to emotional distress. A prospective longitudinal study design would be recommended to bridge this gap. In addition, a pitfall of these approaches is the focus on questionnaires and scoring systems because of the reliance on a cut-off points intended to ´establish´ a phenomenon and socially desirable or exaggerated answers of the respondents. Besides quantitative research, in-depth semi-structured interviews are required to stress the deeper driving forces in an individual to provide more insights into the thoughts and behaviors in reaction to a stressful work environment.

To develop adequate preventive strategies for emotional distress, it is essential to know the individual´s incentive to choose a caring profession in addition to ones unconsciously chosen coping strategies to deal with the stressful work settings. Some encouraging preventive strategies to combat emotional distress in ICU professionals have been developed recently [53,65,80]. A review study of intervention programs for BO found that most of the person-directed interventions, such as cognitive behavioural training, counselling, and relaxation exercises, led to a significant reduction in BO lasting for at least 6 months after the intervention. Although the organization-directed interventions, such as primary nursing, management skills, and social support, were classified by this study as having less study evidence, they were also significantly effective [87]. Combined person- and organization-directed multifaceted interventions with refresher courses reported the best results. At this point, it might be interesting to investigate the effect of a combination of relevant and changeable determinants, such as communication skills, educational sessions in stress management, and mindfulness training for ICU professionals.

The improvement of communications skills might support the interaction with patients and relatives, and reduce conflicts with colleagues or management [7,64]. An intensive three-day training for oncologists resulted in the integration of many of the key communication skills in their daily practice, for up to 15 months post-course [88]. Furthermore, significantly more expressions of empathy were reported in this study and successively interpreted as an increase of self-efficacy. This, in turn, could enhance compassionate care and increase personal well-being. Educational sessions in stress management might expand the awareness of emotional distress and methods to apply in response to this distress [45,58,62]. The awareness of stressful situations and knowing the vital signs of BO or CF, are the first steps in maintaining a healthy work life. In a lack of awareness the ongoing devastating process may continue until a total mental or physical breakdown. Personality [39] and emotional intelligence [62], especially the meta-cognitive capacity of the individual, might provide some clues for the energy in trying to change things in the ´here and now at the bedside´ within their level of responsibility. Mindful meditation might be a source of strength for preventing the hidden effects of stress, and gives the individual healthcare professional the ability to pay attention in the present moment and respond wisely, instead of reacting later with negative feelings [89,90]. Balancing human intimacy and professional distance, and remaining appropriately present and compassionate, may be recognized as a valuable personal ability. This ability could be taught, and effectively enhanced, through self-awareness and mindful meditation which is potentially useful in promoting well-being and stress management in healthcare professionals [89–91].

### Strengths and Methodological Limitations

The main strengths of this review were the systematic approach and reproducible method. It was based on explicit search strategies, eight applicable databases and unambiguous criteria for selecting suitable and high-quality studies. Because randomized controlled trials or rigorous observational studies are rare in this area, a meta-analysis could not be performed [35].

Although measures in the included studies have been taken to prevent social desirability (e.g. guaranteed anonymity), the internal validity might be threatened due to self-report questionnaires. Furthermore, the Hawthorne effect could have biased the results of the reported studies. Some of these studies tried to limit this bias by explicitly not mentioning the measured concept to the respondents [44,60]. Moreover, the response rates in the very low ends, e.g. selection bias, and high ends, e.g. mandatory participation, could be questioned.

This literature review aimed to be highly sensitive in order to be as comprehensive as possible, and therefore had a lower precision. Thus, many irrelevant references were included in the beginning of the review process. This could lead to an erroneous exclusion of a relevant reference. Further, the search was limited to original articles, which suggests the potential to miss information on the topic. However, because of the focus on the prevalence rather than the causes or consequences, this approach was a justified decision. The restriction in language could also have caused an incomplete overview of the relevant studies.

As in every review, a publication bias may have occurred. Positive results are more likely to be submitted and published in scientific journals than inconclusive or negative results, and insignificant outcomes will probably not be mentioned in an abstract [92,93], accordingly putting too much emphasis on the significant mental effects of stress in the ICU. Moreover, negative or inconclusive results remain unpublished; consequently, there might be an over reported prevalence of burnout or compassion fatigue.

## Conclusions

Working in the ICU environment is an emotionally charged challenge, and the emotional price of caring might become a burden for professionals’ personal lives, possibly manifested in compassion fatigue or burnout. This study adds some new viewpoints in the lack of common understanding of the theoretical constructs, which is reflected by the variously defined (and interpreted) negative outcomes of providing care in the ICU setting among the included studies. The true magnitude of the emotional distress in the ICU healthcare professionals remains unclear due to a lack of unity in measurements as well. This study also suggests that policymakers should introduce interventions to prevent the negative consequences of emotional distress. A longitudinal experimental study is needed to examine the emotional distress among ICU professionals in relation to their communication skills, educational sessions on stress management, and mindfulness. Only in this way evidence-based best practice interventions can be formulated.

## Supporting Information

S1 File PRISMA 2009 Checklist. (DOC)

S1 Table Search protocol. (DOCX)

S2 Table Assessment of articles. (DOCX)
