# Supplementary material for: The Prevalence of Compassion Fatigue and Burnout among Healthcare Professionals in Intensive Care Units: A Systematic Review
Source: PLoS One. 2015 Aug 31;10(8):e0136955. doi: 10.1371/journal.pone.0136955 (PMC4554995; doi:10.1371/journal.pone.0136955)
Supplement: S1 Table — (DOCX) [file pone.0136955.s002.docx]

Search protocol; the prevalence of burnout and compassion fatigue

Number of found references

| Database |  | References | Without duplicates |
| --- | --- | --- | --- |
| Embase |  | 875 | 872 |
| Medline OvidSP |  | 513 | 132 |
| Cinahl |  | 493 | 267 |
| Web-of-science |  | 335 | 165 |
| PsycINFO |  | 138 | 47 |
| PubMed publisher |  | 12 | 11 |
| Cochrane |  | 14 | 6 |
| Google Scholar |  | 200 | 120 |
| **Total ^*^** |  | **2580** | **1620** |

**^*^** Results of systematic searching in electronical databases until 30 June, 2014

**Search strategies**

*Embase 872*

('posttraumatic stress disorder'/de OR (('emotional attachment'/de OR empathy/de OR 'emotional stress'/de OR 'nurse patient relationship'/de OR 'doctor patient relation'/de OR 'coping behavior'/de OR 'runaway behavior'/de OR 'stress management'/de OR 'adaptive behavior'/de) AND (fatigue/de OR exhaustion/de OR burnout/de OR Workload/de OR 'job stress'/de)) OR (((compass* OR empath* OR vicarious OR attach* OR emotion* OR coping OR runaway) NEAR/6 (fatigue* OR satisf* OR stress OR trauma* OR exhaust* OR burnout* OR tired*)) OR ((secondar* OR posttraumat* OR 'post traumatic') NEAR/3 stress)):ab,ti) AND ((('health care personnel'/exp OR nursing/exp OR 'health personnel attitude'/exp OR (personnel* OR nurs* OR doctor* OR physician* OR caregiver* OR provider* OR paramedic* OR profession* OR staff):ab,ti) AND ('intensive care'/exp OR 'intensive care unit'/de OR ('intensive care' OR 'critical care' OR icu OR picu OR nicu OR icus OR picus OR nicus):ab,ti)) OR 'intensive care nursing'/exp )

*Medline OvidSP 132*

("Stress Disorders, Post-Traumatic"/ OR ((empathy/ OR "Stress, Psychological"/ OR exp "Professional-Patient Relations"/ OR "Adaptation, Psychological"/ OR "runaway behavior"/) AND (exp fatigue/ OR "Burnout, Professional"/ OR "Workload"/)) OR (((compass* OR empath* OR vicarious OR attach* OR emotion* OR coping OR runaway) ADJ6 (fatigue* OR satisf* OR stress OR trauma* OR exhaust* OR burnout* OR tired*)) OR ((secondar* OR posttraumat* OR "post traumatic") ADJ3 stress)).ab,ti.) AND (((exp "health personnel"/ OR exp nursing/ OR nursing.xs. OR exp "Attitude of Health Personnel"/ OR exp "Professional Role"/ OR (personnel* OR nurs* OR doctor* OR physician* OR caregiver* OR provider* OR paramedic* OR profession* OR staff).ab,ti.) AND (exp "Critical Care"/ OR exp "intensive care units"/ OR ("intensive care" OR "critical care" OR icu OR picu OR nicu OR icus OR picus OR nicus).ab,ti.)))

*Cochrane 6*

((((compass* OR empath* OR vicarious OR attach* OR emotion* OR coping OR runaway) NEAR/6 (fatigue* OR satisf* OR stress OR trauma* OR exhaust* OR burnout* OR tired*)) OR ((secondar* OR posttraumat* OR 'post traumatic') NEAR/3 stress)):ab,ti) AND ((((personnel* OR nurs* OR doctor* OR physician* OR caregiver* OR provider* OR paramedic* OR profession* OR staff):ab,ti) AND (('intensive care' OR 'critical care' OR icu OR picu OR nicu OR icus OR picus OR nicus):ab,ti)) )

*Web-of-science 165*

TS=(((((compass* OR empath* OR vicarious OR attach* OR emotion* OR coping OR runaway) NEAR/6 (fatigue* OR satisf* OR stress OR trauma* OR exhaust* OR burnout* OR tired*)) OR ((secondar* OR posttraumat* OR "post traumatic") NEAR/3 stress))) AND ((((personnel* OR nurs* OR doctor* OR physician* OR caregiver* OR provider* OR paramedic* OR profession* OR staff)) AND (("intensive care" OR "critical care" OR icu OR picu OR nicu OR icus OR picus OR nicus)))))

*PsycINFO OvidSP 47*

("Posttraumatic Stress Disorder"/ OR ((empathy/ OR "Psychological Stress"/ OR "Adaptation"/ OR "runaway behavior"/) AND (exp fatigue/ OR "Occupational Stress"/ OR "Work load"/)) OR (((compass* OR empath* OR vicarious OR attach* OR emotion* OR coping OR runaway) ADJ6 (fatigue* OR satisf* OR stress OR trauma* OR exhaust* OR burnout* OR tired*)) OR ((secondar* OR posttraumat* OR "post traumatic") ADJ3 stress)).ab,ti.) AND (((exp "health personnel"/ OR exp nursing/ OR "Health Personnel Attitudes"/ OR (personnel* OR nurs* OR doctor* OR physician* OR caregiver* OR provider* OR paramedic* OR profession* OR staff).ab,ti.) AND (exp "intensive care"/ OR ("intensive care" OR "critical care" OR icu OR picu OR nicu OR icus OR picus OR nicus).ab,ti.)))

*CINAHL* 267

(MH "Stress Disorders, Post-Traumatic+" OR ((MH empathy+ OR MH "Stress, Psychological+" OR MH "Professional-Patient Relations+" OR MH "Adaptation, Psychological+") AND (MH fatigue+ OR MH "Burnout, Professional+" OR MH "Workload+")) OR (((compass* OR empath* OR vicarious OR attach* OR emotion* OR coping OR runaway) N6 (fatigue* OR satisf* OR stress OR trauma* OR exhaust* OR burnout* OR tired*)) OR ((secondar* OR posttraumat* OR "post traumatic") N3 stress))) AND (((MH "health personnel+" OR MH "Attitude of Health Personnel+" OR MH "Professional Role+" OR (personnel* OR nurs* OR doctor* OR physician* OR caregiver* OR provider* OR paramedic* OR profession* OR staff)) AND (MH "Critical Care+" OR MH "intensive care units+" OR ("intensive care" OR "critical care" OR icu OR picu OR nicu OR icus OR picus OR nicus))) OR MH "Critical Care Nursing+")

*PubMed publisher 11*

((((compass*[tiab] OR empath*[tiab] OR vicarious[tiab] OR attach*[tiab] OR emotion*[tiab] OR coping[tiab] OR runaway[tiab]) AND (fatigue*[tiab] OR satisf*[tiab] OR stress[tiab] OR trauma*[tiab] OR exhaust*[tiab] OR burnout*[tiab] OR tired*[tiab])) OR ((secondar*[tiab] OR posttraumat*[tiab] OR post traumatic[tiab]) AND stress[tiab]))) AND ((((personnel*[tiab] OR nurs*[tiab] OR doctor*[tiab] OR physician*[tiab] OR caregiver*[tiab] OR provider*[tiab] OR paramedic*[tiab] OR profession*[tiab] OR staff)) AND ((intensive care[tiab] OR critical care[tiab] OR icu[tiab] OR picu[tiab] OR nicu[tiab] OR icus[tiab] OR picus[tiab] OR nicus[tiab])))) AND publisher[sb]

*Google Scholar 120*

"compassion|empathy|vicarious|attachment|emotional fatigue|satisfaction|stress|exhaustion"|ptss|ptsd personnel|nursing|nurses|doctors|physicians|caregivers|providers|paramedics|professional|staff icu|picu|nicu|icus|picus|nicus
