# Supplementary material for: The Prevalence of Compassion Fatigue and Burnout among Healthcare Professionals in Intensive Care Units: A Systematic Review
Source: PLoS One. 2015 Aug 31;10(8):e0136955. doi: 10.1371/journal.pone.0136955 (PMC4554995; doi:10.1371/journal.pone.0136955)
Supplement: S2 Table — (DOCX) [file pone.0136955.s003.docx]

Assessment of articles

**Box 1. Inclusion criteria**

| *Inclusion criteria first selection round on title abstract:* | |
| --- | --- |
|  | Studies dealing with the prevalence of burnout, compassion fatigue, vicarious trauma or secondary traumatic stress, and intervention studies on these kind of emotional distress. |
|  | Studies in the Intensive Care Unit, Critical Care Unit, Neonatology Intensive Care Unit, Pediatric Intensive Care Unit, |
|  | Studies on nurses or physicians, |
|  | Studies published from 1992 until 23 May 2014, |
| *Inclusion criteria second selection round after whole article inspection:* | |
|  | Studies in English (or Dutch) language, |
|  | Original and review articles available in complete form |
| *Inclusion criteria on methodological soundness:* | |
|  | Prevalence defined as percentages of burnout or compassion fatigue |
|  | At least six of eight criteria on soundness must be available:   - Research questions and objectives are described precisely - Clear definition of compassion fatigue - A valid and reliable measuring instrument - Method is described in detail - Information given on size and type of the target populations - Information given on number and characteristics of subjects who agreed to participate - Drop out/missing values are addressed - Statistical analysis appropriate |

Table 1.

*References included/excluded after whole text reading*

| # | Include/exclude | Author(s) | Year | Title of article | Journal |
| --- | --- | --- | --- | --- | --- |
| Studies dealing with the prevalence of burnout, compassion fatigue, vicarious trauma or secondary traumatic stress | | | | | |
| 1 | Include | Galvan, M. E., J. C. Vassallo, et al. | 2012 | Physician's burnout in pediatric intensive care units from Argentina | Arch Argent Pediatr 110(6): 466-473 |
| 2 | Include | Barbosa, F. T., B. A. Leao, et al. | 2012 | Burnout syndrome and weekly workload of on-call physicians: Cross-sectional study. | Sao Paulo Med J 130(5): 282-288. |
| 3 | Exclude, not prevalence | Rama-Maceiras, P., S. Parente, et al. | 2012 | Job satisfaction, stress and burnout in anesthesia: Relevant topics for anesthesiologists and healthcare managers? | Eur J Anaesthesiol 29(7): 311-319 |
| 4 | Include | Bellieni, C. V., P. Righetti, et al. | 2012 | Assessing burnout among neonatologists | J Matern -Fetal Neonatal Med 25(10): 2130-2134. |
| 5 | Include | Shehabi, Y., G. Dobb, et al. | 2008 | Burnout syndrome among Australian intensivists: a survey. | Crit Care Resusc 10(4): 312-315. |
| 6 | Exclude, other work setting | Kholdebarin, R., R. M. Helewa, et al. | 2011 | Evaluation of a regional acute care surgery service by residents in general surgery. | J Surg Educ 68(4): 290-293. |
| 7 | Include | Embriaco, N., E. Azoulay, et al | 2007 | High level of burnout in intensivists: prevalence and associated factors. | Am J Resp Crit Care Med 175(7): 686-692 |
| 8 | Exclude, other work setting | Chiron, B., E. Michinov, et al. | 2010 | Job Satisfaction, Life Satisfaction and Burnout in French Anesthetists. | J Health Psych 15(6): 948-958. |
| 9 | Exclude, other work setting | Shanafelt, T. D., C. M. Balch, et al. | 2010 | Burnout and Medical Errors Among American Surgeons | Annals of Surgery 251(6): 995-1000. |
| 10 | Include | Elkonin, D. and Lizelle | 2011 | Positive and negative emotional responses to work-related trauma of intensive care nurses in private health care facilities. | Health SA Gesondheid 16(1): 1-8 |
| 11 | Exclude, only fatigue not CF | Ruggiero, J. S | 2003 | Correlates of fatigue in critical care nurses. | Res Nurs Health 26(6): 434-444 |
| 12 | Exclude, not prevalence | Todaro-Franceschi, V. | 2013 | Critical care nurses' perceptions of preparedness and ability to care for the dying and their professional quality of life | DCCN 32(4): 184-190 |
| 13 | Exclude, not prevalence | Marcial, L., M. Brazina, et al. | 2013 | A brief article: is this the cost of caring? A student perspective on compassion fatigue. | Dim Crit Care Nurs 32(1): 18-21. |
| 14 | Include | Mealer, M. L., A. Shelton, et al. | 2007 | Increased prevalence of post-traumatic stress disorder symptoms in critical care nurses. | Am J Respir Crit Care Med 175(7): 693-697 |
| 15 | Include | Raftopoulos, V., A. Charalambous, et al. | 2012 | The factors associated with the burnout syndrome and fatigue in Cypriot nurses: a census report. | BMC Public Health 12 |
| 16 | Exclude, not prevalence | Smart, D., A. English, et al. | 2013 | Compassion fatigue and satisfaction: A cross-sectional survey among US healthcare workers. | Nurs Health Sci |
| 17 | Include | Su, T. P., T. C. Lien, et al. | 2007 | Prevalence of psychiatric morbidity and psychological adaptation of the nurses in a structured SARS caring unit during outbreak: A prospective and periodic assessment study in Taiwan. | J Psychiatr Res 41(1-2): 119-130 |
| 18 | Include | Young, J. L., D. M. Derr, et al. | 2011 | Compassion satisfaction, burnout, and secondary traumatic stress in heart and vascular nurses. | Crit Care Nurs Q 34(3): 227-234 |
| 19 | Exclude, not prevalence | Sluiter, J. K., A. P. Bos, et al. | 2005 | Is staff well-being and communication enhanced by multidisciplinary work shift evaluations? | Intens Care Med 31(10): 1409-1414 |
| 20 | Exclude, not prevalence | Meadors, P. and A. Lamson | 2008 | Compassion Fatigue and Secondary Traumatization: Provider Self Care on Intensive Care Units for Children. | J Pediatr Health Care 22(1): 24-34 |
| 21 | Include | Czaja, A. S., M. Moss, et al. | 2012 | Symptoms of Posttraumatic Stress Disorder Among Pediatric Acute Care Nurses. | J Pediatr Nurs 27(4): 357-365 |
| 22 | Exclude, not prevalence | Ayala, E. and A. M. Carnero | 2013 | Determinants of Burnout in Acute and Critical Care Military Nursing Personnel: A Cross-Sectional Study from Peru. | PLoS ONE 8(1). |
| 23 | Include | Liu, Y. E., A. While, et al. | 2013 | Job satisfaction and work related variables in Chinese cardiac critical care nurses. | J Nurs Manag |
| 24 | Include | Teixeira, C., O. Ribeiro, et al. | 2013 | Ethical decision making in intensive care units: a burnout risk factor? Results from a multicenter study conducted with physicians and nurses. | J Med Ethics |
| 25 | Exclude, not prevalence | Losa Iglesias, M. E. and R. Becerro de Bengoa Vallejo | 2013 | Prevalence and relationship between burnout, job satisfaction, stress, and clinical manifestations in behavior critical care nurses. | DCCN 32(3): 130-137. |
| 26 | Exclude, not prevalence | Epp, K. | 2012 | Burnout in critical care nurses: a literature review. | Dynamics 23(4): 25-31. |
| 27 | Include | Karanikola, M. N., E. D. Papathanassoglou, et al. | 2012 | Burnout syndrome indices in Greek intensive care nursing personnel.” | DCCN 31(2): 94-101. |
| 28 | Include | Goetz, K., S. Beutel, et al. | 2012 | Work-related behavior and experience patterns of nurses.” | Int Nurs Rev 59(1): 88-93. |
| 29 | Include | Liu, K., L.-M. You, et al. | 2012 | The relationship between hospital work environment and nurse outcomes in Guangdong, China: a nurse questionnaire survey. | J ClinNursi 21(9/10): 1476-1485. |
| 30 | Exclude, not prevalence | Klopper, H. C., S. K. Coetzee, et al. | 2012 | Practice environment, job satisfaction and burnout of critical care nurses in South Africa. | J Nurs Manage 20(5): 685-695. |
| 31 | Include | Merlani, P., M. Verdon, et al. | 2011 | Burnout in ICU caregivers: a multicenter study of factors associated to centers.” | Am J Resp Crit Care Med 184(10): 1140-1146. |
| 32 | Exclude, not original article | Azoulay, E. and M. Herridge | 2011 | Understanding ICU staff burnout: The show must go on. | Am J Respir Crit Care Med 184(10): 1099-1100. |
| 33 | Include | Rochefort, C. M. and S. P. Clarke | 2010 | Nurses’ work environments, care rationing, job outcomes, and quality of care on neonatal units. | J Adv Nurs 66(10): 2213-2224. |
| 34 | Include | Cho, S., K. J. June, et al. | 2009 | Nurse staffing, quality of nursing care and nurse job outcomes in intensive care units. | J ClinNurs 18(12): 1729-1737. |
| 35 | Include | Verdon, M., P. Merlani, et al. | 2008 | Burnout in a surgical ICU team. | Intens Care Med 34(1): 152-156. |
| 36 | Include | Lederer, W., J. F. Kinzl, et al. | 2008 | Fully developed burnout and burnout risk in intensive care personnel at a university hospital. | Anaesth Intens Care 36(2): 208-213. |
| 37 | Include | Raggio, B. and P. Malacarne | 2007 | Burnout in Intensive Care Unit. | Minerva Anestesiol 73(4): 195-200. |
| 38 | Include | Poncet, M. C., P. Toullic, et al. | 2007 | Burnout syndrome in critical care nursing staff. | Am J Resp Crit Care Med 175(7): 698-704. |
| 39 | Exclude, not original article | Embriaco, N., L. Papazian, et al. | 2007 | Burnout syndrome among critical care healthcare workers. | Curr Opin Crit Care 13(5): 482-488. |
| 40 | Exclude, not prevalence | Bakker, A. B., P. M. Le Blanc, et al. | 2005 | Burnout contagion among intensive care nurses. | J Adv Nurs 51(3): 276-287. |
| 41 | Exclude, not prevalence | Buhler, K. E. and T. Land | 2004 | Burnout and personality in extreme nursing: An empirical study. | Schweiz Arch Neurol Psychiatr 155(1): 35-42. |
| 42 | Exclude, not prevalence | Chen, S. M. and A. McMurray | 2001 | “Burnout” in intensive care nurses.” | J Nurs Res 9(5): 152-164. |
| 43 | Exclude, not prevalence | Tekindal, B., M. A. Tekindal, et al. | 2012 | Nurses’ burnout and unmet nursing care needs of patients’ relatives in a Turkish State Hospital. | Int J Nurs Pract 18(1): 68-76. |
| 44 | Include | Quenot, J. P., J. P. Rigaud, et al. | 2012 | Suffering among carers working in critical care can be reduced by an intensive communication strategy on end-of-life practices. | Intens Care Med 38(1): 55-61. |
| 45 | Exclude, not prevalence | Cubrilo-Turek, M., R. Urek, et al. | 2006 | Burnout syndrome – Assessment of a stressful job among intensive care staff | Coll Antropol 30(1): 131-135. |
| 46 | Exclude, not prevalence | Kerasiotis, B. and R. W. Motta | 2004 | Assessment of PTSD symptoms in emergency room, intensive care unit, and general floor nurses. | Int J Emerg Ment Health 6(3): 121-133 |
| 47 | Exclude, not prevalence | Ozden, D., S. Karagozoglu, et al. | 2013 | Intensive care nurses’ perception of futility: Job satisfaction and burnout dimensions. | Nurs Ethics 20(4): 436-447. |
| 48 | Exclude, case study | Pardoe, P. | 2011 | Psychological support for nurses on pediatric intensive care units | Nurs Child Young People 23(8): 27-29 |
| 49 | Exclude, not prevalence | Ifeagwazi, R. F. | 2005 | The influence of marital status on self-report of symptoms of psychological burnout among nurses. | Omega: J Death Dying 52(4): 359-373. |
| 50 | Include | Meadors, P., A. Lamson, et al. | 2010 | Secondary traumatization in pediatric healthcare providers: Compassion fatigue, burnout, and secondary traumatic stress | Omega J Death Dying 60(2): 103-128 |
| 51 | Include | Guntupalli, K. K.  Fromm, r. E. | 1996 | Burnout in the internist-intensivist | Intens Care Med 22: 625-630 |
| 52 | Exclude, not prevalence | Aytekin, A., et al. | 2013 | Burnout levels in neonatal intensive care nurses and its effects on their quality of life. | Australian Journal of Advanced Nursing 31(2): 39-47. |
| 53 | Include | Guntupalli, K. K., et al. | 2014 | Burnout in the intensive care unit professionals. | Indian J Crit Care Med 18(3): 139-143. |
| 54 | Exclude, not prevalence | Myhren, H., et al. | 2013 | Job Satisfaction and Burnout among Intensive Care Unit Nurses and Physicians | Crit Care Res Pract 2013: 786176. |
| 55 | Include | Saini, R., et al. | 2011 | Assessment of stress and burnout among intensive care nurses at a tertiary care hospital | J. Mental Health Human Behav 16(1) 43-48 |
| 56 | Include, same data as # 24 | Teixeira, C., et al. | 2013 | Burnout in intensive care units - a consideration of the possible prevalence and frequency of new risk factors: A descriptive correlational multicentre study." | BMC Anesthesiol 13:38 |
| 57 | Include | Zhang, X. C., et al. | 2014 | Job burnout among critical care nurses from 14 adult intensive care units in northeastern China: a cross-sectional survey. | BMJ Open 4(6): e004813. |
| 58 | Include | Mason, V. M., et al. | 2014 | Compassion fatigue, moral distress, and work engagement in surgical intensive care unit trauma nurses: a pilot study. | DCCN 33(4): 215-225. |
| 59 | Exclude, not prevalence | Neville, K. and D. A. Cole | 2013 | The relationships among health promotion behaviors, compassion fatigue, burnout, and compassion satisfaction in nurses practicing in a community medical center. | JONA 43(6) 348-354 |
| 60 | Exclude, not prevalence | Mealer, M. and J. Jones | 2014 | Methodological and ethical issues related to qualitative telephone interviews on sensitive topics. | Nurse Res. 21: 32-37. |
| 61 | Exclude, not prevalence | Lin, F., et al. | 2009 | Burnout among hospital nurses in China | J. Nurs Manag 17 294-301 |
| Intervention studies on emotional distress | | | | | |
| 62 | Include, comparison of two groups | Ali, N. A., et al. | 2011 | Continuity of care in intensive care units: a cluster-randomized trial of intensivist staffing. | Am J Respir Crit Care Med 184(7): 803-808. |
| 63 | Exclude, not effect of intervention measured | Caine, R. M. and L. Ter-Bagdasarian | 2003 | Early identification and management of critical incident stress. | Crit Care Nurse 23(1): 59-6 |
| 64 | Include, comparison of two groups | Garland, A | 2012 | Twenty-four-Hour Intensivist Presence A Pilot Study of Effects on Intensive Care Unit Patients, Families, doctors, and Nurses | Am J Respir Crit Care Med 185 (7) 738-743. |
| 65 | Exclude, abstract | Hartshorn, J. C. | 1992 | Evaluation of a critical care nursing internship program | J Cont Educ Nurs 23(1): 42-48. |
| 66 | Exclude, abstract | Iskra-Golec, I., et al. | 1996 | Health, well-being and burnout of ICU nurses on 12- and 8-h shifts. | Work Stress 10(3): 251-256. |
| 67 | Include, pre- and post-measurement | Loiselle, C. G., et al. | 2012 | A pre-post evaluation of the Adler/Sheiner Programme (ASP): a nursing informational programme to support families and nurses in an intensive care unit (ICU). | Intens Crit Care Nurs 28(1): 32-40. |
| 68 | Exclude, abstract | Schwarzkopf, D., et al. | 2012 | A survey of ICU medical staff on interdisciplinary communication about END-of-life care and burnout. | Intens Care Med38: S70-S71. |
| 69 | Include, pre- and post-measurement (Nr 19 first review, not prevalence) | Sluiter, J. K., et al. | 2005 | Is staff well-being and communication enhanced by multidisciplinary work shift evaluations? | Intens care Med 31(10): 1409-1414. |
| 70 | Include, pre- and post-measurement | Beumer, C. M. | 2008 | Innovative solutions: the effect of a workshop on reducing the experience of moral distress in an intensive care unit setting | Dimens Crit Care Nurs 27(6): 263-267. |
| 71 | Include, pre- and post-measurement | Eagle, S., et al. | 2012 | The effect of facilitated peer support sessions on burnout and grief management among health care providers in pediatric intensive care units: A pilot study. | J Pall Med 15(11): 1178-1180. |
| 72 | Include, pre- and post-measurement (Nr 20 first review, not prevalence) | Meadors, P. and A. Lamson | 2008 | Compassion Fatigue and Secondary Traumatization: Provider Self Care on Intensive Care Units for Children. | J Pediatr Health Care 22(1): 24-34. |
| 73 | Include, pre- and post-measurement | Mehrabi, T., et al. | 2012 | The effect of yoga on coping strategies among intensive care unit nurses. | Iran J Nurs Midwifery Res17(6): 421-424. |
| 74 | Include, pre- and post-measurement | Nooryan, K., et al. | 2011 | The effect of teaching emotional intelligence (EI) items on job related stress in physicians and nurses working in ICU wards in hospitals, Yerevan, Armenia. | Int J Collab Res Intern Med Public Health 3(10): 704-713. |
| 75 | Include, pre- and post-measurement | Nooryan, K., et al. | 2012 | Controlling anxiety in physicians and nurses working in intensive care units using emotional intelligence items as an anxiety management tool in Iran. | International journal of general medicine 5: 5. |
| 76 | Include | West, C. P., et al. | 2014 | Intervention to Promote Physician Well-being, Job Satisfaction, and Professionalism: A Randomized Clinical Trial. | JAMA internal medicine 174(4): 527-533 |
| Manual found | | | | | |
| 77 | Exclude, not ICU | Mealer, M & Jones, J | 2013 | Posttraumatic Stress Disorder in the Nursing Population: A concept Analysis | Nursing Forum, 48(4) |
| 78 | Exclude, not ICU | Nimmo, A & Huggard, P | 2013 | A Systematic Review of the measurement of Compassion fatigue, Vicarious Trauma, and Secondary Traumatic Stress in Physicians | Austral J Disaster Trauma Studies |
| 79 | Exclude, not ICU | Kaschka, W. P. Korczak, D., & Broich, K. | 2011 | Burnout: a Fashionable Diagnosis. | Deutsches Ärzteblatt International 108(46): 781-1 |

Table 2.

*Methodological criteria*

| # | Research questions and objectives are described precisely | Clear definition of concept(s) has been given | A valid and reliable measuring instrument has been used | Method is described in detail | Information given on size and type of the target populations | Information given on number and characteristics of subjects | Drop out/missing values are addressed | Statistical analysis are appropriate | Total amount of criteria on soundness |
| --- | --- | --- | --- | --- | --- | --- | --- | --- | --- |
| 1 | Y | Y | Y | Y | Y | Y | Y | Y | 8 |
| 2 | Y | Y | Y | Y | Y | Y | N | Y | 7 |
| 4 | Y | Y | Y | Y | Y | Y | Y | Y | 8 |
| 5 | Y | Y | Y | Y | Y | N | Y | Y | 7 |
| 7 | Y | Y | Y | Y | Y | Y | Y | Y | 8 |
| 10 | Y | Y | Y | Y | Y | Y | N | Y | 7 |
| 14 | Y | N | Y | Y | Y | Y | Y | Y | 7 |
| 15 | Y | Y | Y | Y | Y | Y | Y | Y | 8 |
| 17 | Y | N | Y | Y | Y | Y | Y | Y | 7 |
| 18 | Y | Y | Y | Y | Y | N | N | Y | 6 |
| 21 | Y | Y | Y | Y | Y | Y | Y | Y | 8 |
| 23 | Y | N | Y | Y | Y | Y | Y | Y | 7 |
| 27 | Y | N | Y | Y | Y | Y | Y | Y | 8 |
| 28 | Y | Y | Y | Y | Y | Y | Y | Y | 8 |
| 29 | Y | N | Y | Y | Y | Y | Y | Y | 7 |
| 31 | Y | Y | Y | Y | Y | Y | Y | Y | 8 |
| 33 | Y | Y | Y | Y | Y | Y | Y | Y | 8 |
| 34 | Y | N | Y | Y | Y | Y | Y | Y | 7 |
| 35 | Y | Y | Y | Y | Y | Y | Y | Y | 8 |
| 36 | Y | Y | Y | Y | Y | Y | Y | Y | 8 |
| 37 | Y | Y | Y | Y | Y | Y | Y | Y | 8 |
| 38 | Y | Y | Y | Y | Y | Y | N | Y | 7 |
| 44 | Y | Y | Y | Y | Y | Y | Y | Y | 8 |
| 50 | Y | Y | Y | Y | Y | Y | N | Y | 7 |
| 51 | Y | Y | Y | Y | Y | Y | N | Y | 7 |
| 53 | Y | Y | Y | N | Y | Y | N | Y | 6 |
| 55 | Y | N | Y | Y | Y | Y | N | Y | 6 |
| 56 | Y | Y | Y | Y | Y | Y | Y | Y | 8 |
| 57 | Y | Y | Y | Y | Y | Y | Y | Y | 8 |
| 58 | Y | Y | Y | Y | Y | Y | N | Y | 7 |
| 62 | Y | N | N (not for burnout) | Y | Y | Y | Y | Y | 6 |
| 64 | Y | N | Y | Y | Y | Y | Y | Y | 7 |
| 67 | Y | N | Y | Y | Y | Y | Y | Y | 7 |
| 69 | Y | N | Y | Y | Y | Y | Y | Y | 7 |
| 70 | Y | Y | N | Y | Y | N | Y | N | 5 |
| 71 | Y | N | Y | Y | Y | Y | Y | Y | 7 |
| 72 | Y | Y | Y | Y | Y | Y | N | Y | 7 |
| 73 | Y | N | Y | Y | Y | Y | N | Y | 6 |
| 74 | Y | Y | Y | Y | Y | Y | N | Y | 7 |
| 75 | Y | Y | Y | Y | Y | Y | N | Y | 7 |
| 76 | Y | Y | Y | Y | Y | Y | Y | Y | 8 |
